# A bright future for micro-LED displays

**DOI:** 10.1038/s41377-024-01683-z

**Published:** 2024-12-06

**Authors:** Vineeth K. Bandari, Oliver G. Schmidt

**Affiliations:** 1 Research Center for Materials, Architectures and Integration of Nanomembranes (MAIN), Chemnitz University of Technology, Chemnitz, Germany; 2 Material Systems for Nanoelectronics, Chemnitz University of Technology, Chemnitz, Germany; 3 International Institute for Intelligent Nanorobots and Nanosystems (IIINN), Fudan University, Shanghai, China

**Keywords:** Displays, Inorganic LEDs, Photonic devices

## Abstract

The development of GaN-based Micro-LED arrays achieving brightnesses exceeding 10^7^ nits and high-density micro-displays with up to 1080×780 pixels marks a true breakthrough in the field. This breakthrough is a result of mastering a combination of long-standing challenges comprising wafer-scale high-quality epitaxial growth, sidewall passivation, efficient photon extraction, and elegant bonding technologies, and promises significant advantages for augmented and virtual reality devices, wearables, and next-generation consumer electronics.

## Introduction

In the rapidly evolving world of display technologies, innovations in light-emitting diodes (LEDs) continue to push the boundaries of what’s possible in terms of brightness, resolution, and power efficiency. Among the most promising of these are GaN-based Micro-LEDs, a technology that offers superior brightness and color accuracy compared to organic LEDs (OLEDs) and liquid crystal displays. Micro-LEDs are considered the next frontier in visual interfaces, particularly for augmented reality (AR), virtual reality (VR), and high-definition micro-displays. A critical challenge in this field, however, has been the development of green Micro-LEDs, which are essential for accurate color reproduction. Achieving the high brightness and efficiency required for practical use in these devices has proven difficult, especially as pixel sizes shrink below 10 μm. This issue is part of a broader phenomenon known as the “green gap,” where the performance of green LEDs lags behind that of their blue and red counterparts. Studies such as those by Zhang et al.^1^, Qi et al.^2^ and Yu et al.^3^ have made progress in this area, with reported pixel densities of up to 848 pixels per inch (ppi) and significant improvements in external quantum efficiencies^1–3^. In a recent study, Pan et al. have made a remarkable breakthrough in this field by developing ultra-high brightness green Micro-LEDs using wafer-scale uniform GaN epilayers on silicon substrates achieving pixel densities as high as 3400 ppi and brightness levels exceeding 10^7^ nits (cd/m^2^)^4^. This achievement marks a major step forward, offering a solution that overcomes key technical barriers while enabling high-resolution, high-brightness displays suitable for next-generation consumer electronics and wearable applications.

## The Breakthrough

### Challenges Overcome

One of the primary hurdles in the development of green Micro-LEDs has been the efficiency and brightness of small-pixel devices. When Micro-LED pixels are reduced to sizes smaller than 10 μm, issues like sidewall damage from the fabrication process and limited light extraction efficiency arise. These problems lead to reduced luminous efficiency and non-uniform brightness across the display, which are critical drawbacks for applications requiring high-definition visuals. Moreover, the “green gap” has been a longstanding challenge in the field of LEDs. This gap refers to the difficulty in achieving high efficiency in green light-emitting devices, largely due to the poor quality of the materials typically used to produce green light. Key challenges include phase separation during the growth of the epilayers and the quantum-confined Stark effect, both of which reduce the radiative recombination rate and thus the overall brightness of the device. The current work improves on these limitations by a combination of clever growth techniques, surface treatments and bonding technologies, allowing for better photon extraction and higher external quantum efficiency, resulting in brightness levels exceeding 10⁷ nits for GaN-based Micro-LED arrays^4^.

### Key Technical Innovations

**Fig. 1:**
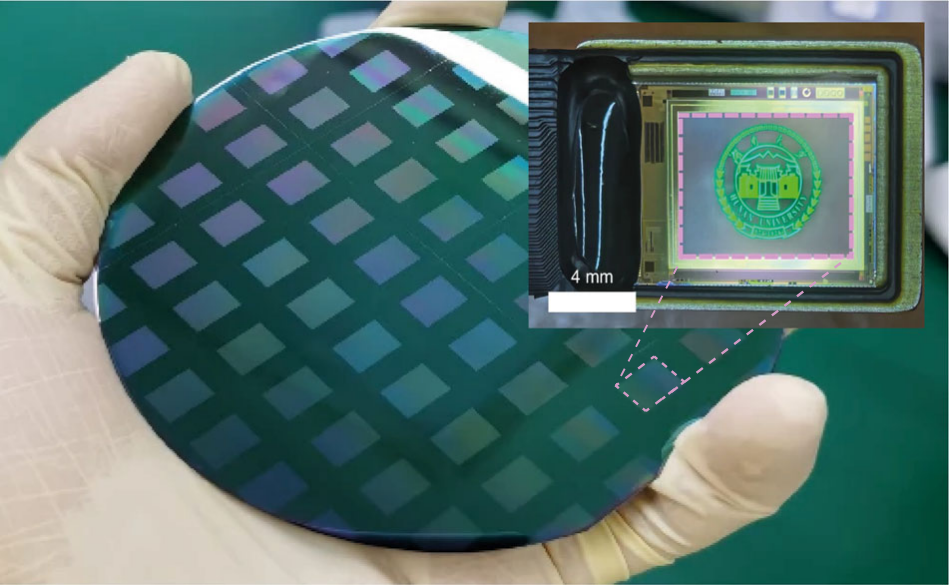
Wafer-scale fabrication of high brightness, high density (3400 ppi) GaN-on-Si Micro-LED displays with 1080 × 780 pixels^4^

The recent breakthrough reported in the study centers around combinig several key innovations in the field into integrated high-performance display technology. The first is the use of high-quality GaN epilayers grown on silicon substrates. Silicon offers an intriguing solution for device integration and scalability. However, the lattice and thermal mismatches between GaN and silicon have historically led to issues like wafer bowing and high dislocation density, both of which degrade device performance. By surfactant mediated growth and careful strain engineering, the researchers successfully grew 4-inch and 6-inch GaN-on-silicon epilayers with a dislocation density as low as 5.25 × 10⁸ cm⁻^2^ and a wafer bow of only 16.7 μm—remarkable metrics that enable fabrication of high-performance Micro-LED arrays^4^. This achievement is complemented by atomic sidewall passivation^5^ that uses potassium hydroxide to smooth the sidewalls of the Micro-LED pixels, followed by the application of a 50 nm Al_2_O_3_ layer using atomic layer deposition. This process not only repairs defects introduced during the etching process but also enhances photon extraction efficiency by creating nanoscale surface textures on the pixel top, allowing for brighter and more uniform light emission. As a result, the team achieved a brightness of over 10⁷ nits, which is the highest reported for GaN green Micro-LEDs on-silicon to date. Finally, the integration of the Micro-LEDs with silicon-based CMOS circuits by an elegant non-alignment bonding technology allowed for the creation of a high-resolution, high-brightness, high density display with up to 1080 × 780 pixels (see Fig. 1).

## Applications and Impact

### Display Technologies

The development of ultra-high brightness Micro-LEDs with wafer-scale uniform GaN-on-silicon epilayers opens up new possibilities for display technologies, especially in areas where brightness, resolution, and power efficiency are crucial. One of the most promising applications of this technology is in AR and VR devices, which require displays capable of delivering high brightness and sharp imagery even under challenging lighting conditions. Current AR/VR displays rely heavily on OLEDs or mini-LED backlighting, which are unable to match the brightness and durability of Micro-LEDs. The ability of these GaN-based Micro-LEDs to achieve over 10^7^ nits makes them a prime candidate for use in these devices, offering users a more immersive and vibrant experience. Additionally, Micro-LEDs are known for their longer lifespan and lower power consumption compared to OLEDs, which often suffer from burn-in and other longevity issues. The high resolution achieved through the integration of Micro-LEDs with silicon CMOS circuits, as demonstrated in this work, further enhances their potential for next-generation displays. With a pixel density reaching up to 3400 ppi and the ability to fabricate displays with 1080 × 780 resolution, this technology could revolutionize not only AR/VR headsets but also applications in wearable technology, automotive displays, and advanced mobile devices.

### Scalability and Cost Efficiency

The scalability of GaN-on-silicon technology is another significant advantage of this research^4^. By using silicon as a substrate, the researchers have demonstrated that it is possible to grow large-scale (4-inch and 6-inch) epilayers with high uniformity and minimal wafer bowing. This advancement improves upon the limitations seen in previous works, where sapphire’s high cost and smaller size restricted mass production. Moreover, silicon-based manufacturing is well established in the semiconductor industry, making it easier to integrate Micro-LEDs with other silicon-based technologies, such as CMOS circuits The vertical non-alignment bonding technique used in this study also represents a significant improvement as it simplifies the fabrication process, reduces alignment errors and further enhances the efficiency of production. The combination of scalability, cost efficiency, and performance makes GaN-on-silicon Micro-LEDs a strong contender for large-scale consumer applications.

### High-Performance Micro-Displays

One of the most exciting applications of this technology is in micro-displays, where pixel sizes are reduced to the micron scale. The ability to achieve uniform brightness and high resolution in small-sized pixels is a critical requirement for AR/VR applications, head-up displays and other near-eye display systems. In this study, the research team’s achievement of a pixel size as small as 5 μm, combined with a pitch size of 7.5 μm, positions this technology as a leader in the race for the next generation of micro-displays. The integration of these Micro-LEDs with CMOS circuits also offers an important advantage: the potential for active matrix displays. This architecture allows for more precise control of individual pixels, enabling higher refresh rates and improved image quality. With their ultra-high brightness and pixel precision, GaN-on-silicon Micro-LEDs could provide the perfect solution for high-definition displays in small, portable devices. Additionally, the high pixel density and uniform brightness achieved in this study open up possibilities for applications in AR headsets, which require compact displays that can deliver bright, clear images in all lighting conditions. Similarly, wearable devices such as smart glasses could benefit from the combination of low power consumption and high performance that these Micro-LEDs offer.

## Conclusion

The development of ultra-high brightness green Micro-LEDs using wafer-scale uniform GaN-on-silicon epilayers marks a major milestone in the field of micro-display technology. By overcoming key technical challenges such as low-quality growth on Si, sidewall damage and poor light extraction efficiency, the researchers have created a highly scalable and cost-effective solution that could pave the way for the widespread adoption of Micro-LEDs in various industries. With their unprecedented brightness, high resolution, and integration with silicon-based circuits, these Micro-LEDs are poised to stir up display industry, enabling new possibilities in AR/VR, wearable tech, and beyond. The ability to achieve over 10⁷ nits of brightness and integrate with CMOS circuits positions this technology as a front-runner in the race for high-performance, energy-efficient displays, not only for consumer electronics but also for automotive, industrial, and medical applications.

## References

[1] Zhang, X. et al. High-resolution monolithic micro-LED full-color micro-display. *SID Symp. Dig. Tech. Pap.* **51**, 339–342 (2020).

[2] Qi, L. H. et al. 848 ppi high-brightness active-matrix micro-LED micro-display using GaN-on-Si epi-wafers towards mass production. *Opt. Express* **29**, 10580–10591 (2021). 33820191 10.1364/OE.419877

[3] Yu, J. C. et al. Gallium nitride blue/green micro-LEDs for high brightness and transparency display. *IEEE Electron Device Lett.* **44**, 281–284 (2023).

[4] Wu, H. F. et al. Ultra-high brightness Micro-LEDs with wafer-scale uniform GaN-on-silicon epilayers. *Light Sci. Appl.* **13**, 284 (2024). 39384779 10.1038/s41377-024-01639-3 PMC11464674

[5] Yan, X. et al. Enhanced light extraction efficiency of GaN-based green micro-LED modulating by a thickness-tunable SiO_2_ passivation structure. *Opt. Express* **31**, 39717–39726 (2023). 38041287 10.1364/OE.506590

